# Persistent elevation of plasma markers of cellular senescence after hip fracture: a pilot longitudinal study

**DOI:** 10.3389/fragi.2024.1477528

**Published:** 2024-11-12

**Authors:** Eric J. Lenze, Ginger E. Nicol, George A. Kuchel, Michael S. Avidan, Breno S. Diniz

**Affiliations:** ^1^ Department of Psychiatry, Washington University School of Medicine, St. Louis, MO, United States; ^2^ UConn Center on Aging, UConn Health Center & School of Medicine, Farmington, CT, United States; ^3^ Department of Anesthesiology, Washington University School of Medicine, St. Louis, MO, United States

**Keywords:** senescence-associated secretory phenotype, cellular senescence, hip fracture, biology of aging, aging

## Abstract

**Introduction:**

Hip fractures may result from and contribute to accelerated biological aging. We aimed to evaluate the impact of hip fracture and its surgery on the senescence-associated secretory phenotype (SASP) index, a composite of peripheral protein markers where higher scores are thought to indicate greater levels of cellular senescence and accelerated aging.

**Methods:**

We examined the SASP index in plasma over 12 weeks post-surgery and its prediction of long-term post-surgical functional outcomes. We included 60 older adults: 20 recruited immediately after hip fracture surgery, and 40 comparison individuals who were either healthy or suffering chronic psychosocial stress (caregiving). We assessed 22 SASP biomarkers and calculated the SASP index score for each hip fracture participant immediately following fracture surgery and 4 and 12 weeks later. Functional recovery was assessed at 12, 26, and 52 weeks after hip replacement surgery.

**Results:**

The hip fracture group had higher SASP index scores than the comparison groups, after adjusting for potential confounding variables (*p* = 0.021). SASP index scores in hip fracture patients increased further by week 4 after surgery (*p* < 0.001), declining by week 12 but remaining elevated overall. However, the SASP index scores were not significantly associated with functional recovery after hip replacement surgery at 26 or 52 weeks after surgery. In conclusion, after hip fracture surgery SASP scores are elevated, continue to rise over time, and do not return to normal by 12 weeks post-surgery.

**Discussion:**

Our findings support the need to investigate this phenomenon of post-operative senescence, including whether novel interventions such as senolytics would help older adults facing major surgery.

## Introduction

The study of biological aging processes has gained increasing scientific interest for studying accelerated aging. Hip fracture is a common (Veronese and Maggi, 2018) and serious condition in older adults and may reflect accelerated aging because of functional decline and premature mortality post-fracture (Bliuc et al., 2013). Hip fractures often require surgical repair or replacement. Both the fracture and the surgery are major physiological stressors and can trigger an acute inflammatory response (Oelsner et al., 2017; Poredos et al., 2021; Reikeras et al., 2014; Watt et al., 2015; Moghtadaei et al., 2019) with a persistent elevation of inflammatory cytokines over 4–7 days after surgical procedure (Sadahiro et al., 2020). Hip fracture is also a psychological stressor, with high levels of perceived stress, depression, and anxiety, which can persist for weeks or months after surgery (Kornfield et al., 2017; Lenze et al., 2007). Thus, hip fracture surgery is a combined physiological and psychological stressor, with inflammation suggested to have a key role (Matheny et al., 2011).

The Geroscience hypothesis posits that the biological processes of aging are the primary underlying cause of many chronic diseases that are common with aging (Kennedy et al., 2014). It is based on the existence of hallmarks of biological aging, such as chronic pro-inflammatory, cellular senescence, DNA damage, among others (Lopez-Otin et al., 2023). Abnormalities in these processes can lead to accelerated biological aging and higher risk of premature aging phenotypes. Surgical stress and related inflammatory challenges offer an opportunity to examine prevention and targeted treatment approaches for the acceleration in age-related biological changes that follow surgery, particularly in older adults. A recent study evaluated the impact of surgical trauma on DNA methylation (DNAm)-based biological aging clocks (Poganik et al., 2023). They found that biological age measured by second-generation DNAm aging clocks (e.g., GrimAGE) increased immediately (i.e., 24 h) after emergency and elective hip replacement surgery and decreased 4–7 days later. However, this study did not assess biological aging parameters over a longer period (weeks to months).

Cellular senescence is one of the main hallmarks of biological aging (Gasek et al., 2021; Kuehnemann and Wiley, 2024). Post-operative cellular senescence changes play a fundamental role in normal wound healing (He and Sharpless, 2017). However, an excessive or prolonged cellular senescence response has been linked to adverse health outcomes and poorer prognosis in distinct medical disorders (Schafer et al., 2020; Sauver et al., 2023). One of the key features of cellular senescence is the change of the cellular secretory pattern, called the senescence-associated secretory phenotype (SASP) (Sharpless and Sherr, 2015; Wang et al., 2024). The SASP can be measured in the blood and is thought to indicate the cellular senescence burden at a given time (Basisty et al., 2020). In this study, we evaluated the impact of hip fracture surgery on the SASP over 12 weeks post-operatively. We also evaluated if the SASP is associated with short and long-term functional recovery (up to 52 weeks) after surgery. We hypothesized that SASP index scores would be elevated after hip fracture surgery, but would return to baseline levels 12 weeks after surgery. We also hypothesized that higher SASP index scores would predict worse functional recovery 26 and 52 weeks after hip fracture surgery.

## Methods

This was a prospective longitudinal study examining the effects of a combined physiological and psychological stressor (hip fracture surgery) on mood, cognitive functioning and SASP index in older adults. Twenty adults aged 60+ with hip fracture (the “Hip Fracture” group) requiring surgical repair were enrolled from hospitals in Saint Louis. Forty comparison participants were enrolled from the community in two groups: 20 healthy comparisons (i.e., individuals with no hip fracture in the prior year and no severe medical event or hospitalization in the previous 6 months), and 20 caregivers for partners with dementia who were similarly physically healthy but self-reported a moderate to high level of chronic psychosocial stress (the “Caregiver” group).

Key exclusion criteria for the Hip Fracture group included history of cognitive impairment (i.e., documented history of clinical dementia or delirium that did not clear within 1–2 days of hip surgery, evidenced by a Short Blessed Test score >12), current major depression immediately prior to hip fracture, the presence of metastatic cancer, or taking depressogenic or pro-inflammatory/anti-inflammatory medications (e.g., interferon or corticosteroids). The Washington University institutional review board approved the study, and all participants provided written informed consent.

At baseline, the Cumulative Illness Rating Scale for Geriatric (CIRS-G) score was used to assess medical illness burden. Cognitive function was examined by the Repeatable Battery for the Assessment of Neuropsychological Status (RBANS), a measure of global cognitive functioning (standardized so that mean age-adjusted cognitive performance = 100 with standard deviation = 15); the RBANS was administered at week 4 post-surgery. Comparison groups completed the same baseline measures as the hip fracture participants. Depressive symptoms measured via the Montgomery-Asberg Depression Rating Scale (MADRS) a clinical interview of current (past week) symptom severity where a score ≥10 is consistent with clinically significant depression). RBANS and MADRS scores were available for Hip fracture participants over 52 weeks with assessments conducted in person at baseline (i.e., week 0: 2–3 days after surgery and while still hospitalized), as well as weeks 4 and 52 after discharge (otherwise by phone). The Hip Fracture Recovery (HFR) score (Zuckerman et al., 2000), which assesses basic and instrumental activities of daily living and mobility (complete independence results in a score of 100%), was used to measure functional recovery and was conducted at weeks 0, 4, 12, 26, and 52.

### Senescence-associated secretory phenotype (SASP) factors

Blood was collected by venipuncture at baseline (immediately post-surgical hospitalization) and 4 and 12 weeks in Hip Fracture patients. Individuals in the Control and Caregiver groups had blood drawn at baseline only. Blood was collected with K2-EDTA tubes after overnight fasting and processed immediately after collection by centrifugation at 3,000 g for 10 min at 4°C. Plasma was separated, aliquoted and stored in a −80°C freezer until laboratory analysis with a customized multiplex LUMINEX platform assay (R&D system, MN, United States). All experiments were performed according to the manufacturer’s instructions. All blood biomarkers were analyzed using the same assay batch; the coefficient of variation (CV) was <10% for all analytes. All samples were analyzed on the day of collection to reduce variability across laboratory experiments.

The SASP factors included in the SASP index are listed in the Supplementary Table 1. We selected the biomarkers included in the present analysis based on preclinical studies focused on the secretome observed in senescent cells (Coppé et al., 2008) and our previous analysis of the same proteomic markers in older depressed adults (Seitz-Holland et al., 2023; Diniz et al., 2022). The raw data were log2 transformed and standardized to the z-score. The SASP index score for each participant was calculated based on the following regression formula:

SASP index=β1x1+…+β22x22

where β is the individual weight and x is the standardized value of each biomarker included in the SASP index. The weight for each factor was derived using an independent and clinically heterogeneous sample of older adults with and without a history of MDD (Diniz et al., 2022). The SASP index mean was centered at 0, with a standard deviation of 1 in the whole sample.

### Statistical analysis

We carried out analysis of variance (ANOVA) to test for differences in demographic, clinical, and SASP index scores among individuals in the hip fracture, caregiver, and healthy control groups. Next, we used a repeated measure ANOVA to test for within-individuals differences in the trajectory of SASP index scores changes between baseline, 4, and 12 weeks after hip fracture surgery. Additional contrast analyses were done to test for pairwise differences in the SASP index scores between each follow-up timepoint. Finally, we used a linear regression model to test if SASP index scores at week 12 was associated with functional recovery at weeks 12, 26, and 52 after surgery.

## Results

There were no statistically significant differences in age, sex distribution, body mass index between individuals who underwent hip fracture surgery, healthy comparisons, and caregiver groups (Table 1). However, the burden of comorbid medical illness and cognitive dysfunction was more severe in the hip fracture group than in the other groups, whereas individuals in the caregiver group presented with greater depressive symptom severity.

**TABLE 1:** Baseline characteristics of hip fracture, caregiver and healthy comparison groups.

		Groups				
		Healthy comparison	Caregiver	Hip fracture	Statistics	*p*-value
Gender	M	36%	27%	36%	Chi^2^_(2)_ = 1.38	0.5
	F	24%	39%	37%		
Age (mean ± SD)		77.8 ± 7.7	76.3 ± 6.8	76.6 ± 7.9	F_(2,57)_ = 0.20	0.82
BMI (mean ± SD)		26.3 ± 4.4	25.7 ± 3.7	25.6 ± 4.1	F_(2,57)_ = 0.14	0.86
CIRS-G (mean ± SD)		8.6 ± 3.8	10.2 ± 3.3	11.7 ± 4.3	F_(2,57)_ = 3.24	0.046
MADRS (mean ± SD)		2.9 ± 2.4	12.5 ± 8.6	3.0 ± 3.7	F_(2,57)_ = 18.81	<0.001
RBANS (mean ± SD)		98.8 ± 11.9	90.0 ± 15.1	83.4 ± 15.4	F_(2,57)_ = 5.25	0.008
SASP index score (mean ± SD)		1.23 ± 0.17	1.24 ± 0.23	1.41 ± 0.23	F_(2,57)_ = 4.81	0.012

BMI, body mass index; CIRS-G, Cumulative Illness Rating Scale–geriatric version; MADRS, Montgomery-Asberg Depression Rating Scale; RBANS, repetitive battery for the assessment of neuropsychological status; SASP, Senescence-Associated Secretory Phenotype.

**FIGURE 1:**
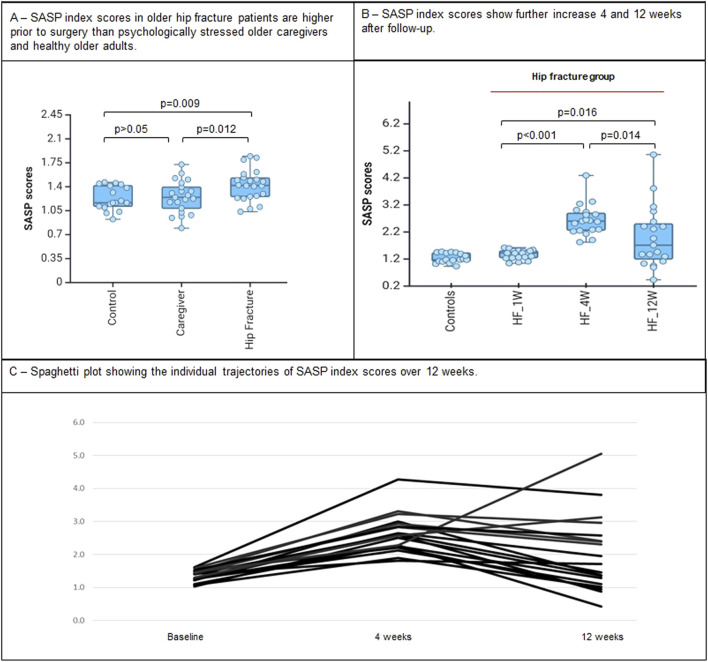
SASP index scores according to baseline groups and its trajectory after hip fracture surgery. **(A)** SASP index scores in older hip fracture patients are higher prior to surgery than psychologically stressed older caregivers and healthy older adults. **(B)** SASP index scores show further increase 4 and 12 weeks after follow-up.

The SASP index scores differed across the three groups at the 4 week time point, with individuals in the Hip Fracture group presenting with the highest SASP index scores, followed by the Caregiver and Healthy Comparison groups (Table 1; Figure 1A). Pairwise contrast analyses showed that the SASP index scores were higher in the Hip Fracture vs Caregiver (*p* = 0.012) and Healthy Comparison groups (*p* = 0.009), but did not differ significantly between Caregiver and Healthy Comparison groups (*p* = 0.78). The association between the SASP index score and Hip Fracture remained statistically significant after controlling for the effect of medical comorbidity burden, depressive symptoms, and cognitive dysfunction (F_2,57_ = 4.15, *p* = 0.021).

Next, we tested the within-individual changes in the SASP index scores 4 and 12 weeks following hip fracture surgery. There was a marked increase in the SASP index scores 4 weeks after hip fracture surgery (estimated marginal difference = 1.28 ± 0.11, *p* < 0.001). After 12 weeks, the SASP index scores declined compared to the 4 week post-surgical time point (estimated marginal difference = −0.64 ± 0.23, *p* = 0.014) but remained significantly higher than the baseline levels (F_1,18_ = 7.13, *p* = 0.016; estimated marginal difference = 0.65 ± 0.24, *p* = 0.016).

Finally, the 12-week post-surgical SASP index score was not significantly associated with 12-week HFR scores (mean = 87.9, std deviation = 10.1; β = −0.11, F = 0.29, *p* = 0.59), nor did the SASP index score predict longer-term functional recovery at post-operative week 26 (mean = 88.1, std deviation = 9.1; β = −0.01, F = 0.16, *p* = 0.69) or week 52 (mean = 91.1, std deviation = 8.2; β = −0.005, F = 0.02, *p* = 0.89).

## Discussion

This study investigated the trajectory of changes in a composite biomarker index of cellular senescence, the SASP index score, in older adults undergoing hip replacement surgery following a hip fracture. This study demonstrated two important results. First, we showed that older adults who had sustained a hip fracture and subsequent hip replacement surgery had markedly elevated SASP index scores, indicating more pronounced systemic senescence changes, compared to healthy older adults and those under chronic psychological stress associated with caring for a loved one with dementia. Second, the highest SASP index score occurred 4 weeks after surgery; however, the SASP index scores at 12 weeks remained significantly higher than the baseline SASP index scores. These results show a long-term effect of surgical trauma on biological processes related to accelerated biological aging, i.e., cellular senescence. These results provide evidence of the long-term effect of surgical trauma on processes related to accelerated biological aging, i.e., cellular senescence. These results are important as hip fracture is associated with premature mortality and may represent a surgical phenotype of accelerated aging.

Recent studies evaluated the impact of surgical trauma on biological aging measures (i.e., biological aging clocks) based on DNA methylation (Poganik et al., 2023; Sadahiro et al., 2020). Although these studies focused on the short-term effects of surgical trauma on the biological aging clocks, they found a significant biological aging acceleration on postoperative day 1 that returned to pre-surgical levels 4–7 days after surgery. In contrast, our work focused on markers related to the cellular senescence secretory phenotype over a longer follow-up period (12 weeks). Our results also showed a significant increase in the SASP that was more pronounced over the first 4 weeks after surgery but did not resume to baseline levels over 12 weeks post-surgery. These findings, together with evidence from the literature, suggest that surgical trauma can acutely affect multiple hallmarks of biological aging (e.g., DNA methylation, cellular senescence, inflammation), but such biological perturbations may have distinct recovery rates after major acute stressors or long-term prognostic value in this population. On the other hand, we cannot exclude that these biological changes are part of physiological response to major surgical trauma, more specifically hip fracture surgery, that promotes healing and functional recovery in this population.

We did not find any association between SASP index scores and functional recovery at 12 weeks after surgery, nor did SASP index scores at 12 weeks predict long-term functional recovery (approximately 6 and 12 months after surgery). These results may indicate that long-term functional recovery after hip replacement surgery is not negatively influenced by the circulating cellular senescence markers. Alternatively, cellular senescence is a key factor influencing earlier phases of wound healing (Andrade et al., 2022). Therefore, we cannot exclude the possibility that the increase in the SASP markers would play a beneficial role in the wound healing process in these individuals and is part of a normal physiological response to acute stressors that support tissue healing and recovery (Magalhães, 2024).

One comparison group was individuals who are undergoing significant psychological chronic stress, i.e., caregivers of patients with dementia. By including individuals under other chronic stress conditions, we could contrast the effect of major acute physiological stressors on a hallmark of biological aging and show its significant impact on markers of cellular senescence. However, previous work from our group showed that psychiatric disorders such as major depressive disorder have been associated with higher SASP score index across the lifespan (Diniz et al., 2019; Diniz et al., 2017). Therefore, one might expect that caregivers with elevated depressive symptoms would have elevated SASP index scores, but our results did not confirm this hypothesis. One possible explanation is that the individuals in the caregiver group were not undergoing psychological stress sufficient to induce accelerated aging, or that adaptative processes were helping them cope with this challenge (Martins et al., 2011).

Our study has important limitations. First, it includes a small sample size with mostly white participants. Thus, further replication of the methods in larger and more diverse populations is necessary. Also, we recruited only older adults undergoing hip fracture surgery, and our findings may not be generalized to other major surgical procedures. On the other hand, the careful longitudinal characterization of the surgical patients, and comparison to non-surgical participants is a strength of our study. Moreover, the SASP biomarkers included in our panel may be overrepresented by inflammatory markers. However, it is well-established that the SASP constituents are mostly pro-inflammatory cytokines (Wang et al., 2024) and thus the overrepresentation of these cytokines are expected in any SASP panel. Also, we acknowledge that there other SASP panel candidates that have been published, for example, IL-10 and TNF-α (Sauver et al., 2023; Schafer et al., 2020) what may have lead to different results, though all SASP panel have many overlapping cytokines. Finally, we focused on the SASP biomarkers as a composite scores instead of focusing on individuals markers. However, the elevation in the SASP index scores in our study is in line with prior studies showing an elevation of individual or few inflammatory biomarkers markers after major surgery (Poredos et al., 2021; Moghtadaei et al., 2019; Watt et al., 2015).

In conclusion, older adults undergoing hip fracture surgery present with elevated markers indicating cellular senescence (SASP index) that further increases by 4 weeks after surgery, and does not return to baseline levels after 12 weeks. However, the elevation of the SASP index was not associated with long-term functional recovery. Future research, including larger sample sizes and more intensive, multimodal biomarkers’ of aging assessments, are necessary to disentangle the role of cellular senescence and SASP on the recovery after hip fracture surgery, and to evaluate their predictive power to identify individuals at higher risk for poor functional and clinical outcomes related to accelerated aging.

## Data Availability

The raw data supporting the conclusions of this article will be made available by the authors, without undue reservation.
