# Supplementary material for: Persistent elevation of plasma markers of cellular senescence after hip fracture: a pilot longitudinal study
Source: Front Aging. 2024 Nov 12;5:1477528. doi: 10.3389/fragi.2024.1477528 (PMC11588694; doi:10.3389/fragi.2024.1477528)
Supplement: Supplementary file 1 [file Table1.docx]

**Supplementary Table 1 – SASP biomarkers and their respective weights to calculate the SASP index scores.**

| **Plasma Protein/Marker** | **Abbreviation** | **SASP index weight** |
| --- | --- | --- |
| Angiogenin | ANG | -0.364 |
| C-C Motif Chemokine Ligand 4 | CCL4 | 0.556 |
| Granulocyte Macrophage Colony-Stimulating Factor | GM-CSF | 0.466 |
| Glycoprotein 130 | Gp130 | 0.403 |
| Growth-related Oncogene alpha | Gro-α | 0.169 |
| Intercellular Adhesion Molecule-1 | ICAM-1 | 0.416 |
| Insulin-like Growth Factor Binding Protein-6 | IGFBP6 | -0.185 |
| Insulin-like Growth Factor Binding Protein-2 | IGFBP2 | -0.104 |
| Interleukin-1 beta | IL-1β | 0.536 |
| Interleukin-6 | IL-6 | 0.364 |
| Interleukin-8 | IL-8 | 0.470 |
| Monocyte Chemotactic Protein-1 | MCP-1 | 0.350 |
| Monocyte Chemotactic Protein-4 | MCP-4 | 0.347 |
| Macrophage Migration Inhibition Factor 1 | MIF-1 | 0.304 |
| Macrophage Inflammatory Protein-1A | MIP-1A | 0.572 |
| Macrophage Inflammatory Protein-3A | MIP-3A | 0.481 |
| Osteoprotegerin | OPG | 0.565 |
| Placental Growth Factor | PLGF | 0.211 |
| Soluble Tumor Necrosis Factor Receptor I | sTNFRI | 0.719 |
| Soluble Tumor Necrosis Factor Receptor II | sTNFRII | 0.612 |
| Tissue Inhibitor Metalloproteinase 1 | TIMP-1 | -0.219 |
| Urokinase-type Plasminogen Activator Receptor | uPAR | 0.583 |
